# Fire needle acupuncture or moxibustion for chronic plaque psoriasis: study protocol for a randomized controlled trial

**DOI:** 10.1186/s13063-019-3736-2

**Published:** 2019-12-04

**Authors:** Zhaoxia Chen, Dongmei Zhou, Yan Wang, Haibing Lan, Xingwu Duan, Bohua Li, Jingxia Zhao, Wei Li, Zhengrong Liu, Tingting Di, Xinwei Guo, Jinchao Zhang, Bo Li, Shuo Feng, Ping Li

**Affiliations:** 1 Beijing Hospital of Traditional Chinese Medicine, Capital Medical University, Beijing, 100010 China; 2 Beijing Institute of Traditional Chinese Medicine, Beijing, 100010 China; 3 Gulou Hospital of Traditional Chinese Medicine of Beijing, Beijing, 100009 China; 4 Dongzhimen Hospital of Beijing University of Chinese Medicine, Beijing, 100700 China; 5 Beijing University of Chinese Medicine, Beijing, 100029 China

**Keywords:** Fire needle therapy, Moxibustion, Plaque psoriasis, Clinical effectiveness, Relapse rate

## Abstract

**Background:**

Psoriasis is a chronic, immune-mediated disorder with chronic plaque psoriasis being the primary manifestation during the remission stage. Patients often have a slow course and long history of the disease. The refractory type of psoriasis is a stubborn rash that does not subside easily. We designed this randomized controlled trial to compare the effectiveness and relapse rates of plaque psoriasis in patients treated with either acupuncture, moxibustion or calcipotriol ointment. The ultimate aim of the study is to select an effective traditional Chinese medicine therapy for patients with plaque psoriasis.

**Methods:**

The study will be a multicenter, prospective, randomized controlled trial that compares the effectiveness of fire needle therapy, moxibustion and calcipotriol ointment. In total, 160 patients with plaque psoriasis who meet the inclusion criteria will be recruited from three hospitals in Beijing and then randomly assigned to receive either fire needle therapy (group A1), moxibustion (group A2) or calcipotriol ointment (group B). All participants will receive an 8-week treatment and will then be followed up for another 24 weeks, with time points at weeks 12 and 24 after treatment completion. The primary outcomes to be measured are relapse rates and psoriasis area and severity index score of the target lesions. In addition, the target lesion onset time, dermatology life quality index, traditional Chinese medicine syndrome score, and the relapse interval of the target lesion will be measured. Adverse events will be recorded for safety assessment.

**Discussion:**

The aim of this study is to determine whether fire needle therapy or moxibustion could improve the clinical effectiveness for psoriasis lesions and reduce the relapse rate. Once completed, it will provide information regarding therapeutic evaluation on fire needle therapy or moxibustion for plaque psoriasis, which will assist clinicians in selecting the most effective treatment options for patients.

**Trial registration:**

International Clinical Trials Registry Platform (ICTRP), ChiCTR1800019588. Registered on 19 November 2018.

## Background

### Epidemiology and current management

Psoriasis is a chronic, immune-mediated disorder with cutaneous and systemic manifestations. It has a severe impact on a patient’s quality of life [1], and an estimated global prevalence of 2–3% [2, 3]. Psoriasis is a polygenic disease and is concurrently observed with rheumatoid arthritis and cardiovascular diseases, especially psoriatic arthritis [4, 5]. The pathophysiology is characterized by abnormal keratinocyte proliferation and immune cell infiltration in the dermis and epidermis involving both the innate and adaptive immune system [6, 7]. The degree of skin damage is variable, with most patients showing disease progression for a few weeks and then relief for a certain period or even remission [8, 9].

Plaque psoriasis is the primary manifestation of psoriasis during remission and is termed the refractory type. Current treatments for moderate to severe plaque psoriasis include topical agents, photo-based therapies, traditional systemic drugs and biologic agents [10]. Biologic therapies that target specific disease mediators have become popular treatment strategies for moderate to severe disease. They are usually bimodal, targeting T cells and inflammatory cytokines, i.e., tumor necrosis factor alpha and interleukin-12/23. At present, there are five biological treatment options approved by the US Food and Drug Administration [11–13], whereas there are limited treatment options for mild to moderate disease.

Although pharmaceutical agents have been effective in rash relief, there are safety concerns with regards to their long-term use due to rash relapse. Apart from the high treatment costs, the adverse events of these treatments include headaches, muscle joint aches, increased risk of cancer [14], infection [15] and lupus [16]. Although calcipotriol ointment is a first-line treatment strategy for moderate plaque psoriasis, it is prone to induce itching, skin irritation, dry skin, erythema, rash and other adverse reactions, and may even induce abnormal blood lipid profiles after prolonged use of large administered doses [17, 18]. Unfortunately, disease relapse is common after drug cessation [19–21]. It has been demonstrated that the recurrence rate of psoriasis was 42.9% following narrow-band ultraviolet B irradiation after 3 months of significant remission, while patients treated with methotrexate had a 73.7% recurrence rate 3 months after treatment [20]. Finding new therapies to prolong remission and prevent disease recurrence are essential to improve the quality of life for patients with psoriasis.

### Rational for the use of intervention

More studies are currently focusing on alternative treatment strategies for psoriasis. These include acupuncture and moxibustion, which have been used in clinical practice as a treatment strategy for psoriasis. Based on the syndrome differentiation of blood stasis due to cold accumulation, for the plaque psoriasis the fire needling therapy and moxibustion can warm channels and expel the cold, tonify Yang-qi and dredge meridians. This will help to remove slough and promote tissue regeneration [22]. Several pilot studies reported that fire needle acupuncture and moxibustion are effective in delaying recurrence compared to conventional therapies [17–19]. These preliminary studies showed their beneficial effect as an adjunct therapy. In the clinical practice, fire needling and moxibustion are readily acceptable in traditional Chinese medicine (TCM) hospitals in China, thus facilitating performing the intervention. Calcipotriol ointment is widely used to resolve the topical lesion of plaque psoriasis [23], which is seen as the first-line therapy [24] and is supported by clinical evidence [25]. This information supports the use of calcipotriol ointment as the standard therapy for a control treatment. This study will compare the effectiveness of the three interventions mentioned above.

### Rationale for the trial design

Currently there is a lack of comparative effectiveness studies using acupuncture-related techniques for plaque psoriasis. Systematic reviews have reported the benefit of needling, but with relatively low methodological quality [26] and a lack of comparison of the effect of different interventions [27]. Furthermore, only a few studies have focused on the relapse rate after the skin lesion subsides [28]. Thus, we designed this prospective randomized controlled trial to determine the clinical effectiveness of fire needling and moxibustion in reducing psoriasis relapse and compared them to calcipotriol ointment, the conventional and most frequently used treatment strategy.

Although the double-blind, placebo controlled trial is the golden standard to assess a therapeutic effect, it is difficult to use this method for the nondrug and manipulation technique of sham needling and moxibustion. With a warm stimulation, the process of manipulation for fire needle and moxibustion is easily distinguished [29]. Thus, blinding and placebo will not be involved in this study. In addition to the outcome measurement choice, this study will also evaluate additional patient-reported outcomes, including target lesion onset time, dermatology life quality index (DLQI), and the TCM syndrome score.

## Methods/design

### Aim, design and setting of the study

The key objectives for this study are to compare the effectiveness and relapse rates of plaque psoriasis in patients treated with either acupuncture, moxibustion or calcipotriol ointment, with a main focus on the relapse rates and psoriasis area and severity index (PASI) score of the target lesions. The ultimate aim of the study is to select an effective TCM clinical therapy for patients with plaque psoriasis.

**Fig. 1:**
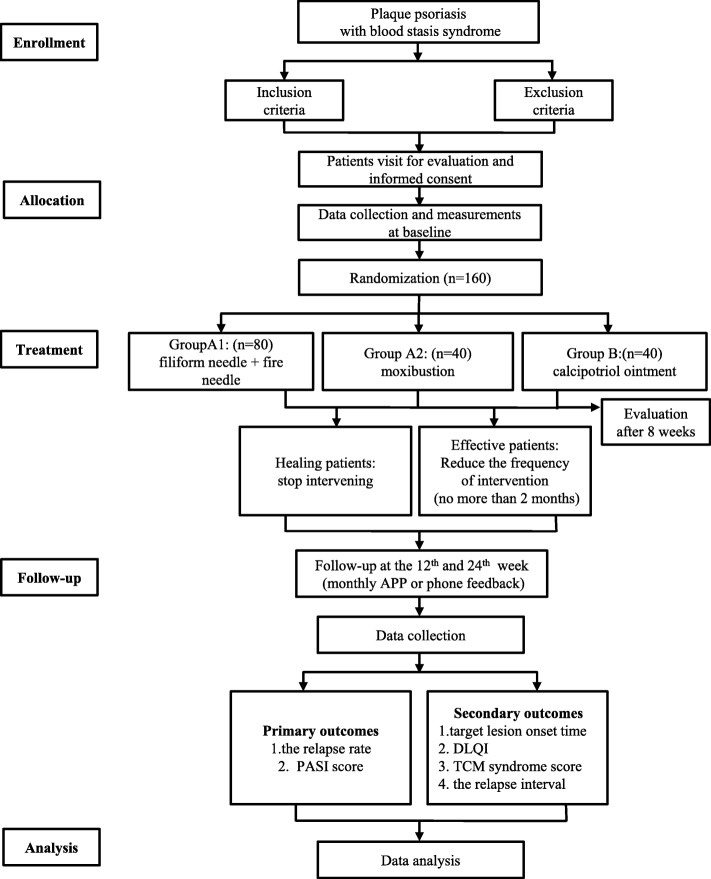
Flowchart of the trial procedure. APP application, DLQI dermatology life quality index; PASI psoriasis area and severity index, TCM traditional Chinese medicine

The proposed study is a multicenter, prospective, randomized controlled trial that will compare the three currently available treatment options for patients with plaque psoriasis. It will consist of an 8-week treatment phase followed by a 24-week follow-up phase. Figure 1 shows the trial procedure and Table 1 details the trial schedule.

**Table 1:**
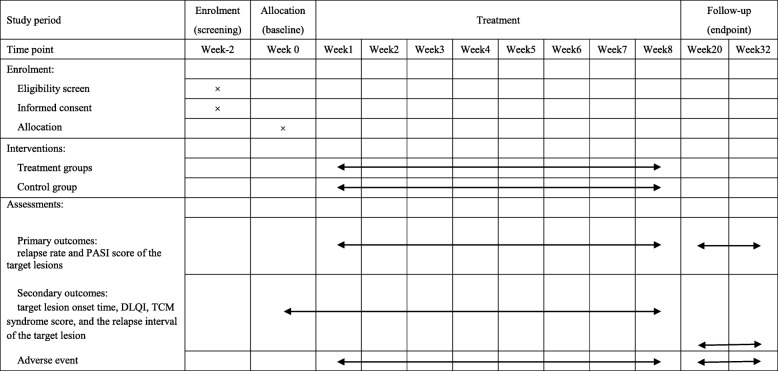
Schedule of enrolment, interventions and assessments

*DLQI* dermatology life quality index; *PASI* psoriasis area and severity index, *TCM* traditional Chinese medicine

Patients will be recruited from the Dermatology Clinic of the Beijing Hospital of Traditional Chinese Medicine, the Capital Medical University, the Dongzhimen Hospital of Beijing University of Chinese Medicine and the Gulou Hospital of Traditional Chinese Medicine of Beijing. All patients will be required to provide written informed consent to participate in the study.

### Eligibility criteria

Patients diagnosed with psoriasis based on the American Dermatology Expert Association criteria will be selected to participate in this study [30].

The inclusion criteria for patient selection are as follows:

- TCM syndrome types with blood stasis.
- No new skin lesions appearing within the last 2 weeks, and lesions are mainly of the plaque type.
- Mild and moderate disease, and the lesion area is no more than 10% of the body surface area.
- Stage of the psoriasis in rest or regression.
- Patients aged between 18 and 65 years old.

Exclusion criteria are as follows:

- Use of glucocorticoids, immunosuppressive drugs, retinoic acid drugs, calcineurin inhibitors, retinoids and/or vitamin D3 derivative preparations in the past month.
- Blood stasis syndrome together with blood heat symptoms.
- Pregnant or lactating women.
- Diagnosed with severe primary disease and mental illness of cardiovascular, cerebrovascular or hematopoietic origin.
- Allergy to the investigational therapies.
- Patients with severe episodes of fainting during acupuncture or afraid of blood.
- Patients participating in other clinical trials.

### Interventions

#### Fire needling

In Group A1, patients will be treated with fire needling. Filiform needles will be used prior to fire needles. This is based on the treatment principles of WeiTong as well as “promoting blood circulation” and “removing blood stasis” using the He’s SanTong method from the Beijing Hospital of Traditional Chinese Medicine.

Fire needling will be conducted in a clinic of the Dermatology Department. The manipulation will mainly be operated on the target lesions. As the target lesions are mostly on the back and limbs of the body, participants will take a prone or sitting position.

**Fig. 2:**
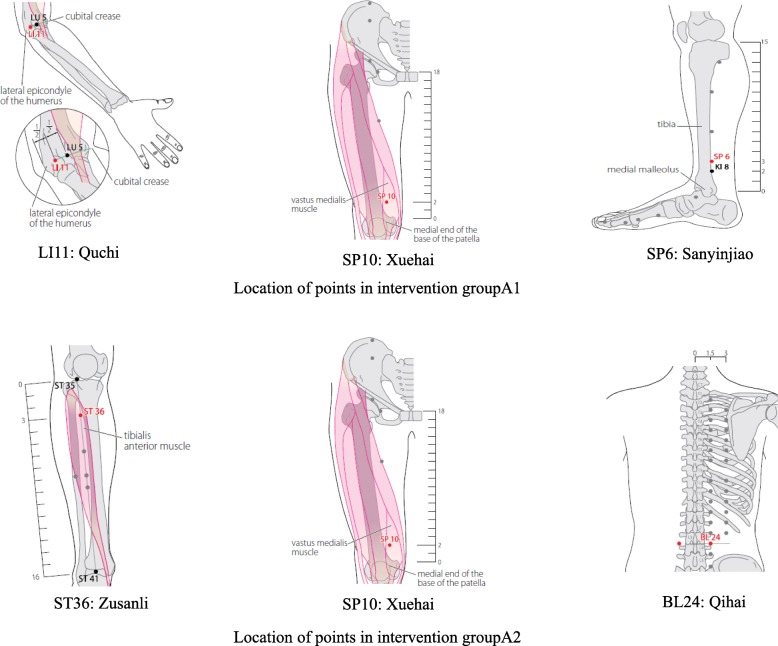
Schematic sites of acupuncture points in the intervention groups A1 and A2. Images are taken from the *WHO standard acupuncture point locations in the western pacific region* [32]

The acupoints used for all participants in the intervention group are shown in Fig. 2. Positioning of the body acupoints will be based on the Chinese–English bilingual innovation textbook of the National College of Advanced Chinese Medicine in the new century “Meridians and Acupoints (Chinese-English)” [31]. The following acupoints (with their locations [32]) will be used:

LI11: On the lateral aspect of the elbow, at the midpoint of the line connecting LU5 (Chize, on the anterior aspect of the elbow, at the cubital crease, in the depression lateral to the biceps brachii tendon) with the lateral epicondyle of the humerus. When the elbow is fully flexed, LI11 is located in the depression on the lateral end of the cubital crease.

SP10: On the anteromedial aspect of the thigh, on the bulge of the vastus medialis muscle, 2 B-cun superior to the medial end of the base of the patella.

SP6: On the tibial aspect of the leg, posterior to the medial border of the tibia, 3 B-cun superior to the prominence of the medial malleolus. 1 B-cun superior to KI8 (Jiaoxin, on the medial aspect of the leg, in the depression posterior to the medial border of the tibial bone, 2 B-cun superior to the prominence of the medial malleolus).

The LI11 (Quchi), SP10 (Xuehai) and SP6 (Sanyinjiao) acupuncture points on both sides of the body will be punctured, with a total of six acupoints per subject.

The acupoints will be punctured one inch perpendicular to the skin surface.

For the filiform needle treatment procedure, the operator will provide lifting, thrusting and rotating to acquire qi.

After acquiring Qi, the needles will be positioned for 20 min.

Needles will be purchased from the Beijing Zhongyan Taihe Medical Equipment Co. Ltd. The dimensions of the filiform needles are 0.25 mm × 40 mm, while the fire needles are 0.5 mm × 40 mm.

Treatment frequency and course will be three times a week for 8 consecutive weeks.

The target lesions will be mostly evaluated from the back and lower limbs. The area of a single lesion should be less than 1% of the body surface area (1 palm size). After treatment with filiform needles, fire needles will be inserted perpendicular to the edge and center of the target lesion. The lesion will be punctured once per 1 cm^2^, at a depth of 4 mm. The lesion area should not be exposed to water for 24 h, and no skin care products should be used.

#### Moxibustion

In Group A2, patients will be treated with moxibustion. The acupoints used for all participants in the intervention group are shown in Fig. 2. This is also based on the treatment principles of WenTong, using the He’s SanTong method from the Beijing Hospital of Traditional Chinese Medicine.

This process will be carried out in a clinic of the Dermatology Department, separately from Group A1. The main region of moxibustion will be selected around the target lesion and the six acupoints. Participants will take a prone or sitting position

The selection principles will be similar to Group A1. The similar acupoints will be ST36 (Zusanli), SP10 and BL24 (Qihai). In addition, the acupoints will be positioned similarly to the previous reference group [24] and Group A1. Acupoints (with their locations [31]) will be positioned as follows:

ST36: On the anterior aspect of the leg, on the line connecting ST35 (Dubi, on the anterior aspect of the knee, in the depression lateral to the patellar ligament) with ST41 (Jiexi, on the anterior aspect of the ankle, in the depression at the center of the front surface of the ankle joint, between the tendons of extensor halluces longus and extensor digitorum longus), 3 B-cun inferior to ST35. ST36 is located on the tibialis anterior muscle.

SP10: On the anteromedial aspect of the thigh, on the bulge of the vastus medialis muscle, 2 B-cun superior to the medial end of the base of the patella.

BL24: In the lumbar region, at the same level as the inferior border of the spinous process of the third lumbar vertebra, 1.5 B-cun lateral to the posterior median line.

The target lesion and all matching acupoints will be treated with moxibustion simultaneously. The baixiao moxibustion device will be put on the target lesion and matched acupoints, with the installed moxibustion column. The moxibustion device is lit and the tube body is rotated to adjust the size of the gas vent to moderate the temperature (generally 42 °C). Each moxibustion column can be applied for 30 min. When the warm feeling disappears and the moxibustion tube is cool, indicating that burning is finished, the device can be removed. After moxibustion, artemisia oil will be used to massage the area for absorption.

Participants with a warm feeling will be seen to achieve the treatment response with moxibustion.

Moxibustion on the target lesion will be performed for 30 min each time, with the matching acupoints treated for 15 min each time.

A baixiao moxibustion device will be used for patient treatment (Chongqing Baixiao Medical Equipment Co. Ltd.), which is composed of the moxibustion cover, positioning paper, moxibustion column, medical adhesive, moxibustion tube and so forth.

Treatment frequency and course will be three times a week for 8 consecutive weeks.

### Control group

Patients in Group B will be treated with calcipotriol ointment. Calcipotriol ointment will be purchased from Leo pharmaceutical Co. Ltd., Ireland (15 g per bottle). Patients will be required to return the used tube for recycling and verification after every visit.

Treatment of the target lesion will be similar to the intervention groups. For each skin lesion (an area the size of the palm), a half-fingertip unit [33] of calcipotriol ointment will be administered. A fingertip unit refers to the dosage of a topical drug that is squeezed from a standard packaging hose with a diameter of 5 mm to an adult fingertip. Calcipotriol ointment will be applied based on the actual lesion area. Treatment frequency and course of intervention for the control group will be once every morning and evening for 8 consecutive weeks.

### Criteria for discontinuing or modifying allocated interventions

The physician will manage the intervention and any related harm for all enrolled patients. Any suspected adverse events related to the treatment will be discussed with the Principal Investigator of the project team. Both the Principal Investigator and physician will be notified of all adverse events and the research team will conduct a quarterly review of all adverse events that may occur during the trial.

Adverse events may occur during the study. Patients who receive fire needling treatment may suffer from fainting, bleeding, body heat, dizziness and so on. Correspondingly, moxibustion treatment may cause scalding injury, allergy, dyspnea, asthma, etc. Topical drugs may cause local redness, irritation, burning, etc. We will establish detailed medical records of the above possible adverse reactions or suspected adverse events and have the necessary treatment measures in place to deal with these adverse events.

If the patient shows any discomfort, changes in their condition, or has any unexpected adverse effects, whether related to the treatments or not, the patient will be instructed to inform the physician. The physician will provide the necessary medical treatment to alleviate any adverse conditions. The physician will determine whether the study treatment is ineffective due to poor effects or for other medical reasons. Patients will be informed of the risks of intervention and possible reasons for adverse effects. The patient may make an appointment with the physician to consider discontinuing or modifying their interventions.

### Sample size estimation

Considering that more participants want to be allocated to fire needle therapy, the ratio of the three groups was set as 2:1:1. The sample size that will be required is based on the following hypothesis: there are differences in the relapse rate of the target lesion between fire needle therapy and calcipotriol. The endpoint of a 12-week disease relapse rate was recorded in our previous observations. The proportion of patients in Group A1 is assumed to be 38%, while it is 67% for Group B [16]. Because of a lack of data on the relapse rate with moxibustion, for the comparison between Group A1 and Group A2, the proportion of patients is also set as 38% versus 67%, thus required information size equal to Group B. The test statistic used is the two-sided *Z* test with pooled variance. The significance level of the test is targeted at 0.05. The power achieved to detect a difference is set as 0.8. Therefore, based on the independent proportions power analysis conducted by an independent statistician via PASS 11.0, approximately 66 participants are required in Group A1 and 33 participants in Group A2 and Group B, respectively. The sample size used in our study was increased by an additional 20% in case of loss to follow-up. Hence, the final sample size was 80 in Group A1, and 40 each in Group A2 and Group B. A total of 160 patients will therefore be included in the study.

### Patient recruitment

Advertisements will be posted in the clinic to recruit patients. The rationale of the study, risks and patient randomization will be explained to all patients who meet the inclusion criteria. Patients will receive a written informed consent document with ample time to understand the associated risks and benefits before being asked to sign it. It will be the responsibility of the investigator to ensure that patients have enough time to properly understand the trial rationale and decide whether to participate in the study. Patients who have signed the informed consent to participate in the study will be randomized and assigned a unique ID number (subcenter code and sequence number).

### Randomization and allocation concealment

Sequence generation will be performed using the PROC PLAN process in SAS 9.4 software (Beijing Hospital of TCM Version; Order Number: 9C1XJD). The numbers for each center will be randomly assigned. The proportion of randomization will be set at 2:1:1 for Group A1 (fire needle combined with acupuncture) to Group A2 (moxibustion) to Group B (calcipotriol). A statistician will then encode the grouping results and seal in an envelope, which will be handed to patients based on their enrollment order. Outcome assessment and statistical analysis will be performed by independent statisticians from the Beijing Institute of Traditional Chinese Medicine who are blinded to the group assignments.

### Blinding

In view of the ease of identifying fire needle procedure and moxibustion, a blinding method could not be used for researchers, patients or outcome evaluators involved in the treatment; hence, only the statisticians were blinded.

### Strategies to improve adherence

The practitioners of the intervention will be the physicians who have a senior title and more than 3 years of clinical experience. This is to ensure that the treatments will be administered consistently. The patient may have the prior to get the subsequent visit to physician in the follow-up time of the study, which is to enhance compliance.

### Relevant concomitant care

Patients in this study will be discouraged from any additional specific complementary treatments related to psoriasis throughout the trial, including herbal medicine, psychotherapy, phototherapy and so forth. If patients need medications such as glucocorticoid drugs and immunosuppression, the relevant information will be recorded on the case report form (CRF).

### Study outcomes

#### Primary outcomes

The primary outcomes will include the relapse rate and PASI score of the target lesions [34, 35]. The relapse rate will be defined as the recurrence of skin lesions after recovery that reaches 50% of the original PASI score, based on the American Academy of Dermatology Expert Association.

#### Secondary outcomes

Secondary outcomes will include target lesion onset time, DLQI, TCM syndrome score, and the relapse interval of the target lesion.

### Data collection

Data will be collected using a CRF. The research medical records and case reports will be completed by the researcher for all enrolled patients. Completed case reports will be reviewed by the clinical monitor, and then handed over to the data administrator for data entry and management.

The following patient information will be collected: general information (including name, gender, birth date, marriage status, time of disease diagnosis and onset), complaint and symptoms, family history, use of pharmaceutical drugs, physical examination, skin lesions, additional symptoms evaluated using the TCM scale, target lesion imaging and scoring, and the DLQI questionnaire. Information regarding adverse events will be recorded during the procedure and on follow-up. Images of skin lesions, PASI scores, TCM syndrome assessment, DLQI, etc. will be performed at patient enrollment and after 2, 4, 6 and 8 weeks of treatment and during the follow-up period (12 and 24 weeks after intervention is completed).

### Patient dropouts

Investigators will have the authority to terminate patient participation at any time if the investigator deems it is in the best interests of the patient. Furthermore, the patient will have the right to withdraw consent to participate in the study at any time for any reason without any consequences for further medical treatment. Patient study discontinuation will be documented.

Termination criteria are as follows: 1) patients with serious adverse event based on the investigator’s judgment will terminate patient study participation; 2) the patient’s condition is aggravated during the course of the disease, and serious complications or rapid deterioration of the condition result in patient treatment discontinuation; 3) other adverse symptoms affecting the study observation or patient wellbeing; 4) unrelated medical reasons; 5) deviations in the clinical trial protocol, such as poor compliance or difficulty in evaluating drug effects; 6) patient thinks there is a lack of effect and voluntarily withdraws; 7) the patient is unwilling to continue clinical treatment during the clinical trial protocol; and 8) patients are administered glucocorticoids and/or immunosuppressive drugs, calcineurin inhibitors, retinoids, and vitamin D3 derivative preparations during the study.

### Study retention

The follow-up observation study protocol is as follows:

1. After 8 weeks of treatment, the patients will enter the follow-up period. Patients who benefited from the treatment will be maintained on the treatment but at a reduced treatment frequency, with treatment not exceeding 2 months.
2. During the follow-up period, patients will be monitored using mobile application software, telephone, SMS text or other methods to communicate on a regular basis. Additionally, patient health management, regular health education and life guidance will be provided. A record of the patient’s medications will be monitored. Patients who benefited from treatment and were still in relapse at the 6-month follow-up will be further evaluated for long-term efficacy. The patient’s recovery will be monitored using application software or telephone feedback information every 4 weeks. Changes in target lesion area will be monitored via application every 2 weeks. Patients will be required to visit the clinic every 3 months to monitor symptoms and signs, conditions, PASI score, skin DLQI and TCM syndrome efficacy score.
3. If the patient relapses, the follow-up will terminate. Patients will then be required to seek medical advice and treatment promptly.

### Data management

A database will be established using EpiData 3.1 software (EpiData Association, Denmark). For the data entry and management, medical statisticians will appoint data administrators. In order to ensure the accuracy of the data, two data entry technicians will independently enter and verify the data twice. Proper training will be provided to the technicians before data entry.

After completion of the data entry, reports on the consistency of the database will be generated. Any inconsistencies found in the database will be verified and corrected.

SAS (version 9.4) software will be used for data verification. Verification will include logical errors, missing data and extreme data. Afterwards, a data verification report will be generated to be sent to the clinical monitor for verification. Any inaccuracies will be checked by the data administrator, who will perform data modification, confirmation, and entry based on the researcher’s response. After both parties are satisfied with the final data version, it will be locked for statistical analysis.

### Statistical analysis

Statistical analysis will be performed using the SAS (version 9.4) software by a statistician who is blinded to the patient grouping. Continuous variables will be presented as mean ± standard deviation (normally distributed data) or medians and ranges (non-normally distributed data). Frequencies and percentages will be used for count data.

Additional statistical tests will be performed using a bilateral differential test, with a *p* value <0.05 considered as statistically significant. For the primary outcome of relapse incidence, survival analysis will be used. Measurement data will be analyzed using *t* test and rank sum test. Count data will be analyzed using chi-square test and Fisher’s exact test, while grade data will be analyzed using Ridit and CMH. Overall evaluation index and the main efficacy indexes will simultaneously be analyzed using per-protocol and intention-to-treat analysis, while multicenter count data will be analyzed using the CMH method. Variance analysis will be performed for measurement data. For confounding factors that are difficult to predict or that are uncontrolled before treatment, i.e., imbalance between the groups before treatment, the least squares mean of covariance analysis and its 95% confidence limit or logistic will be used as covariates. Regression will be used to determine differences in efficacy between the groups and to eliminate the effects of these confounding factors on efficacy.

### Confidentiality of data

Researchers are responsible for maintaining the anonymity of the subjects. Participant information can be identified in the CRF or other documents only by capital letters, numbers and/or code, rather than using the name of the participant. For data storage, research records will be kept safely by researchers of the clinical trial according to the relevant provisions of the Chinese Good Clinical Practice after trial termination for 10 years. After expiration, the researchers will keep data based on the specific circumstances.

### Data monitoring and auditing

In consideration of the intervention not involving pharmaceutical and post-marketing drugs, a for-profit bias would not be involved in this process; the research study team will therefore not engage a data monitoring committee for this study. The research study team will assign a dedicated qualified individual to conduct inspection and entrust a third-party professional institution (the Beijing Clinical Research Quality Promotion Center) not affiliated with the study to conduct regular inspections. This center will prove the data source and ensure data reliability. External inspection will be organized by the Beijing Municipal Health Commission every year. The Beijing Clinical Research Quality Promotion Center will have access to the final trial dataset, and disclosure of contractual agreements that limit such access for investigators.

### Dissemination policy

We will disseminate the results of the study widely through workshops, conference presentations and publications. Once this manuscript is published, a brief summary of results using plain language will be sent to all participants. Authors of the publication should be the directly related researchers of this study.

### Post-trial care

This study will not provide any post-trial care.

### Protocol amendments

If the protocol changes during the implementation of the study, researchers will communicate the important protocol modifications (e.g., changes to eligibility criteria, outcomes, analyses) to the relevant parties, including the fund regulator (Beijing Municipal Health Commission), trial participants, trial registries and journals.

### Roles and responsibility

This study was not sponsored by any pharmaceutical company. It is funded and regulated by Beijing Municipal Health Commission. All the authors participated in the trial design and will collect data and write reports. Being entrusted by the Beijing Municipal Health Commission, the Beijing Clinical Research Quality Promotion Center will play the role of coordinating and steering committee and audit of the study. The Center for Evidence-based Chinese Medicine in Beijing Institute of Chinese Medicine will analyze the collected data and publish the statistical analysis report.

## Discussion

Psoriasis is a chronic, inflammatory, immune-mediated skin disease [36, 37]. The pathogenesis of the disease is complex with many pathogenic factors. These factors may include infection, genetic, environmental, psychological and immune-related. Stress, trauma, alcoholism, and so forth, may be triggering factors [38], and may increase or decrease with age [39]. The disease is characterized by frequent relapses [40] and lingering disease. Psoriasis may not be confined to the skin only; the joints may also be involved, such as both the axial and peripheral joints [41], and it may also be associated with psychological distress [42]. It seriously affects the quality of life and is an economic burden to the patient, their family and society.

At present, Western medicine is used to treat local or systematic disease and can alleviate rash, but is unable to prevent recurrence. The recurrence rate of psoriasis is still relatively high [20, 21]. In addition, the use of Western medicine has several adverse effects. Previous studies have shown that in patients treated with brodalumad (an interleukin-17 targeted biological) the 3-month recurrence rate was 78% [19], while the 48-week recurrence rate in patients treated with ixekizumab was as high as 87% [43]. In addition, the 4-month recurrence rate in patients treated with narrow-spectrum ultraviolet B was 54.55% [44]. Hence, physicians have developed an interest in using simple and low-cost acupuncture and moxibustion methods to treat psoriasis in order to reduce the recurrence rate. From the perspective of TCM, plaque psoriasis at the resting stage is mostly a ‘blood stasis’ syndrome. Acupuncture and moxibustion can promote blood circulation and remove blood stasis, regulate qi and blood circulation, warm meridians and disperse cold. Previous studies have demonstrated that acupuncture and moxibustion have good application prospects for the treatment of plaque psoriasis [22, 28, 45, 46]. In addition, studies have shown that acupuncture has an obvious advantage over Western medicine in reducing the recurrence rate of psoriasis [47]. However, many studies have focused on the combination of multiple treatments simultaneously, such as acupuncture and moxibustion combined with TCM, Western medicine, lasers, and so forth; they have not evaluated the efficacy of acupuncture or moxibustion alone. Hence, this study will evaluate the efficacy of acupuncture or moxibustion for the treatment of plaque psoriasis at the resting stage without the compounding interference of combination treatment strategies. We found that the combination of fire and filiform acupuncture and the simple use of moxibustion has a beneficial effect in the regression of plaque psoriasis in preliminary clinical studies using small patient cohorts.

We hypothesize that acupuncture and moxibustion could improve the effective treatment rate for plaque psoriasis and reduce the recurrence rate and related adverse events. Because of the high incidence and recurrence of psoriasis, current treatment strategies are not ideal. We believe that the results of this study will have important clinical significance, even if our study demonstrates that acupuncture and moxibustion have no clinical efficacy. This will demonstrate that more clinical studies are needed to find a safe and effective treatment strategy for plaque psoriasis. Finding an effective treatment for short- and long-term efficacy is key for psoriasis treatment. This will promote and popularize TCM for psoriasis treatment and other dermatological ailments. This is one of the main benefits of performing the current study.

Several limitations to our research design and study should be noted. First, because of the particularity of the fire needle procedure the method was difficult to blind. The moxibustion method has a risk of scalding and other adverse events. Second, patients visiting TCM hospitals may be inclined to choose TCM methods over Western medicine treatment, i.e., a reluctance to use calcipotriol ointment. This may lead to a lower enrollment rate and longer time to recruit patients for the control group. Third, because of fear of the fire needle procedure, patients may be reluctant to comply with the procedure or volunteer to be enrolled in the experimental group. Finally, the sample cohort was not large, with only patients in Beijing being selected for the study.

In summary, this project will utilize He’s SanTong method from the Beijing Hospital of Chinese Medicine to perform this study. Using this method, we hope to form a comprehensive treatment plan for plaque psoriasis (blood stasis syndrome) to improve clinical efficacy. The recurrence rate and time of long-term efficacy will be observed to determine whether fire acupuncture could further reduce the recurrence rate of psoriasis compared with Western medicine and, in addition, provide evidence for acupuncture treatment of psoriasis. At the same time, we advise that future clinical studies adhere to strict quality controls to fully understand the characteristics of psoriasis, as well as extend follow-up time, focus on the occurrence of adverse reactions, and standardize the use of a subjective PASI scoring standard with a combination of modern skin imaging techniques. Determining these indicators will improve the accuracy and clinical value of the data generated. It will be important to design rigorous, high-quality multicenter, large-cohort, randomized controlled clinical trials and conduct basic research to determine the mechanisms of how acupuncture and moxibustion alleviate psoriasis. Our study should help with standardizing acupuncture and moxibustion treatment strategies for psoriasis.

### Trial status

The trial protocol is version 2.0 at 13 November 2018. This randomized controlled trial began recruiting patients on 1 April 2019. The date when recruitment will be completed is estimated to be December 2020. Active enrollment is open until the required number of patients are enrolled for statistical analysis. A report on the results of our study will be submitted for publication approximately 10 months after data collection and analysis.

## Data Availability

The datasets generated and/or analyzed are available from the corresponding author upon reasonable request.

## Abbreviations

CRF: Case report form
DLQI: Dermatology life quality index
PASI: Psoriasis area and severity index
TCM: Traditional Chinese medicine
